# Supplementary material for: Content-rich biological network constructed by mining PubMed abstracts
Source: BMC Bioinformatics. 2004 Oct 8;5:147. doi: 10.1186/1471-2105-5-147 (PMC528731; doi:10.1186/1471-2105-5-147)
Supplement: Additional File 2 — The original results of the above study (non-essential files are deleted to keep the file size under the limit set by BMC bioinformatics). [file 1471-2105-5-147-S2.bz2 › chilibotAdditionalFile2/dip40/297ID7486683E1288/html/PIK3R1_IRS1.html]

 


 **PIK3R1** and **IRS1** 
  
Found 143 abstracts in PubMed, retrieved 40.  
 

 What does Google say? 
 PDF only 
| .edu only 

---

**Interactive relationship** (e.g. stimulation, inhibition, etc)

**Inhibitory relationship**- Our data show that the PI3K  [ **PIK3R1** ]  activity associated with IRS 1  [ **IRS1** ] , the docking of IRS 1  [ **IRS1** ]  to InR beta and the docking of p85 alpha  [ **PIK3R1** ]  to IRS 1  [ **IRS1** ]  are attenuated in the aged Snell dwarf.  Ref: 12020946 Mech Ageing Dev, 2002
- PDK1, mTOR, and MAPK inhibitors did not block insulin induced reduction of IRS 1  [ **IRS1** ] , suggesting that the PI3K  [ **PIK3R1** ]  serine kinase activity causes IRS 1  [ **IRS1** ]  serine phosphorylation and its commitment to proteasomal degradation.  Ref: 12594228 J Biol Chem, 2003
- Conversely, adenoviral driven expression of constitutively active PI3K  [ **PIK3R1** ]  induced an insulin independent reduction in IRS 1  [ **IRS1** ]  2 protein levels.  Ref: 12594228 J Biol Chem, 2003
- Desensitization was correlated to a reduction in insulin receptor substrate IRS 1  [ **IRS1** ]  and IRS  [ **IRS1** ]  2 protein levels, which was reversed by the PI3K  [ **PIK3R1** ]  inhibitor LY294002.  Ref: 12594228 J Biol Chem, 2003
- RESULTS Chronic treatment of insulin 100 nmol L induced a significant reduction in the phosphorylation and protein expression of IR ?,  **IRS1**  and IRS2, which therefore resulted in a downregulation of association of PI3K  [ **PIK3R1** ]  with IRS  [ **IRS1** ] .  Ref: 12511230 Acta Pharmacol Sin, 2003
- We conclude that 1 PKC alpha is not required for insulin stimulated glucose transport, and 2 PKC alpha is activated by insulin at least partly independently of PI3K  [ **PIK3R1** ] , and largely serves as a physiological feedback inhibitor of insulin signaling to the insulin receptor substrate 1  [ **IRS1** ]  PI3K  [ **PIK3R1** ]  PKB PKC lambda zeta iota complex and dependent metabolic processes.  Ref: 11923480 Mol Endocrinol, 2002
- The MEK1 mutant, which activates ERK, markedly down regulated expression of the insulin receptor IR and its major substrates, IRS 1  [ **IRS1** ]  and IRS  [ **IRS1** ]  2, mRNA and protein, and in turn reduced tyrosine phosphorylation of IR as well as IRS 1  [ **IRS1** ]  and IRS  [ **IRS1** ]  2 and their associated phosphatidyl inositol 3 kinase PI3K  [ **PIK3R1** ]  activity.  Ref: 12554784 Mol Endocrinol, 2003
**Stimulatory relationship**- Finally, the addition of a phosphorylated 14 3 3 binding peptide to an adipocyte homogenate to remove 14 3 3 from IRS 1  [ **IRS1** ]  increased the abundance of IRS 1  [ **IRS1** ]  PI3K  [ **PIK3R1** ]  complexes in the HSP and decreased their abundance in the cytosol.  Ref: 11875115 Mol Endocrinol, 2002
- Co treatment of cells with insulin and LY294002, while reducing total IRS 1  [ **IRS1** ]  phosphorylation, increased its phosphotyrosine content, enhancing IRS 1  [ **IRS1** ]  PI3K  [ **PIK3R1** ]  association.  Ref: 12594228 J Biol Chem, 2003
- The enhancement of IRS 1  [ **IRS1** ]  binding to 14 3 3 by insulin was accompanied by movement of IRS 1  [ **IRS1** ]  and the p85 subunit of PI3K  [ **PIK3R1** ]  from the HSP to the cytosol.  Ref: 11875115 Mol Endocrinol, 2002
- Exposure to IGF 1 for 30 minutes or E2 for 20 minutes increased insulin receptor substrate 1  [ **IRS1** ]  IRS 1  [ **IRS1** ]  association with the regulatory p85 subunit of PI3K  [ **PIK3R1** ] , enhanced tyrosine phosphorylation of p85, and increased PI3K  [ **PIK3R1** ]  activity.  Ref: 12701063 Metabolism, 2003
- A negative role for Ca is also indicated in proximal insulin signaling steps, in that, depletion of intracellular Ca blocks  **IRS1**  serine threonine phosphorylation and enhances insulin stimulated protein protein interaction and PI3K  [ **PIK3R1** ]  activity.  Ref: 11818508 Mol Endocrinol, 2002
- We sought to examine the effects of elevated plasma NEFA approximately 1 mmol liter on muscle PI3K  [ **PIK3R1** ]  activity, insulin receptor substrate IRS 1  [ **IRS1** ]  important for activation of PI3K  [ **PIK3R1** ] , and Akt, which is downstream of PI3K  [ **PIK3R1** ]  and activated by phosphorylation on serine and threonine in a PI3K  [ **PIK3R1** ]  dependent manner.  Ref: 11788651 J Clin Endocrinol Metab, 2002
- Here, we report that insulin induces tyrosine phosphorylation of the insulin receptor and IRS 1  [ **IRS1** ]  and 2, increases binding of activated IRS 1  [ **IRS1** ]  and 2 to the regulatory subunit of PI3K  [ **PIK3R1** ] , and activates protein kinase B Akt, a downstream target of PI3K  [ **PIK3R1** ] .  Ref: 12540590 Diabetes, 2003
- In contrast, there was a high level of IRS  [ **IRS1** ]  2 expression and insulin stimulated tyrosyl phosphorylation as early as embryonic day 15 with robust PI3K  [ **PIK3R1** ]  binding and activation, which may enhance hepatocyte survival during the rapid growth phase of the liver.  Ref: 12891559 Gastroenterology, 2003
- IRS 1  [ **IRS1** ]  over expression in transgenic Tg mouse livers caused constitutive activation of Erk mitogen activated protein kinase MAPK and phosphatidylinositol 3 kinase PI3K  [ **PIK3R1** ]  resulting in significantly increased levels of DNA synthesis and larger hepatic masses relative to non transgenic non Tg littermates.  Ref: 12763374 J Hepatol, 2003
**Neutral relationship**- In contrast, metformin in pharmacological concentration 1 10 mmol L further inhibited tyrosine phosphorylation of IR ?,  **IRS1** , IRS2 and the interaction of PI3K  [ **PIK3R1** ]  with IRS  [ **IRS1** ] .  Ref: 12511230 Acta Pharmacol Sin, 2003
- Tyrosine phosphorylation of insulin receptor substrate 1  [ **IRS1** ]  is detected when enriched by immunoprecipitation with anti PI3K  [ **PIK3R1** ]  antibody.  Ref: 12577306 J Cell Biochem, 2003
- Contrarily, insulin induced IRS  [ **IRS1** ]  2 down regulation occurred via a PI3K  [ **PIK3R1** ]  mTOR pathway.  Ref: 12594228 J Biol Chem, 2003
- Cbl, IRS 1  [ **IRS1** ] , and IRS  [ **IRS1** ]  2 mediate effects of rosiglitazone on PI3K  [ **PIK3R1** ] , PKC lambda, and glucose transport in 3T3 L1 adipocytes.  Ref: 11956152 Endocrinology, 2002
- Using an immunohistochemical technique to detect PI, 5 triphosphate, the main product of PI3K  [ **PIK3R1** ]  activity, we further demonstrate that in the arcuate nucleus, insulin induced PI3K  [ **PIK3R1** ]  activity occurs preferentially within cells that contain IRS  [ **IRS1** ]  2.  Ref: 12540590 Diabetes, 2003

**Non-interactive relationship** (e.g. studied together, co-existance, homology, etc.)

- IRS 1  [ **IRS1** ]  content and insulin responsiveness of PI3K  [ **PIK3R1** ] , Akt, and S6K1 showed a transition to the adult phenotype during the first several postnatal weeks.  Ref: 12217903 Am J Physiol Endocrinol Metab, 2002
- IRS 1  [ **IRS1** ]  association with Grb2 and PI3K  [ **PIK3R1** ]  p85, which link IGFRI to the Ras MAPK and PI3K  [ **PIK3R1** ]  Akt pathways, respectively, was enhanced in both cell types in the presence of EN.  Ref: 12173038 Oncogene, 2002
- Insulin is a potent inducer of adipogenesis, and differentiation of adipocytes requires many components of the insulin signaling pathway, including the insulin receptor substrate IRS 1  [ **IRS1** ]  and phosphatidylinositol 3 kinase PI3K  [ **PIK3R1** ] .  Ref: 12807888 J Biol Chem< MedlineTA>J Biol Chem, 2002
- Importantly, weight loss in obese subjects normalizes PKClambda zeta activation and increases IRS 1  [ **IRS1** ]  phosphorylation and PI3K  [ **PIK3R1** ]  activity.  Ref: 12882908 DiabetesDiabetes, 2002
- The MKK6 mutant, which activates p38, moderately inhibited IRS 1  [ **IRS1** ]  and IRS  [ **IRS1** ]  2 expressions and IRS 1  [ **IRS1** ]  associated PI3K  [ **PIK3R1** ]  activity without exerting a significant effect on the IR.  Ref: 12554784 Mol Endocrinol, 2003
- METHODS We determined the expression and tyrosyl phosphorylation of the insulin receptor beta subunit IRbeta, IRS 1  [ **IRS1** ]  and IRS  [ **IRS1** ]  2, the binding of phosphatidylinositol 3 kinase PI3K  [ **PIK3R1** ] , and activation of the mitogen activated protein kinase MAPK pathway in the presence or absence of insulin stimulation in vivo during development and in the adult liver.  Ref: 12891559 Gastroenterology, 2003
- Shb overexpression resulted also in the assembly and activation of a multiunit complex consisting of at least Shb, IRS 1  [ **IRS1** ] , IRS  [ **IRS1** ]  2, FAK, and PI3K  [ **PIK3R1** ] .  Ref: 12520086 Mol Med, 2002
- Finally, the MKK7 mutant, which activates JNK, reduced tyrosine phosphorylation of IRS 1  [ **IRS1** ]  and IRS  [ **IRS1** ]  2 and IRS  [ **IRS1** ]  associated PI3K  [ **PIK3R1** ]  activity without affecting expression of the IR, IRS 1  [ **IRS1** ] , or IRS  [ **IRS1** ]  2.  Ref: 12554784 Mol Endocrinol, 2003
- In summary, NEFA induced insulin resistance is associated with an impairment of IRS 1  [ **IRS1** ]  tyrosine phosphorylation and IRS 1  [ **IRS1** ]  associated PI3K  [ **PIK3R1** ]  activation.  Ref: 11788651 J Clin Endocrinol Metab, 2002
- Conclusion It is concluded that the Shb induced alterations in the IRS 1  [ **IRS1** ]  PI3K  [ **PIK3R1** ]  Akt pathway may be relevant to the understanding of growth and death patterns of insulin producing cells.  Ref: 12520086 Mol Med, 2002
- Insulin stimulated insulin receptor substrate IRS 1  [ **IRS1** ]  tyrosine phosphorylation and PI3K  [ **PIK3R1** ]  activity are impaired 40 50% in diabetic subjects compared with lean or obese subjects.  Ref: 12882908 DiabetesDiabetes, 2002
- Exposure to 100 nM IGF 1 for 10 min resulted in increased insulin receptor substrate 1  [ **IRS1** ]  associated PI3K  [ **PIK3R1** ]  activity and Akt kinase activity.  Ref: 12624428 Endocrine< MedlineTA>Endocrine, 2002
- The appearance of a complex between tyrosine phosphorylated insulin receptor substrate 1  [ **IRS1** ]  IRS 1  [ **IRS1** ]  and PI3K  [ **PIK3R1** ]  in a high speed pellet fraction HSP is thought to be a key event in insulin action.  Ref: 11875115 Mol Endocrinol, 2002
- The insulin induced increase in IRS 1  [ **IRS1** ]  associated PI3K  [ **PIK3R1** ]  activity was impaired by elevated NEFA, so that activity at the end of the clamps with Intralipid was 35% lower than in the control clamps P <.  Ref: 11788651 J Clin Endocrinol Metab, 2002
- Presently, we found that insulin signaling to insulin receptor substrate 1  [ **IRS1** ]  dependent PI3K  [ **PIK3R1** ] , PKB, and PKC lambda, and downstream processes, glucose transport and activation of ERK, were enhanced in skeletal muscles and adipocytes of mice in which the ubiquitous conventional diacylglycerol sensitive PKC isoform, PKC alpha, was knocked out by homologous recombination.  Ref: 11923480 Mol Endocrinol, 2002
- docking of p 85 alpha to p110 alpha or p110 beta, and IRS  [ **IRS1** ]  2 associated PI3K  [ **PIK3R1** ]  activity.  Ref: 12020946 Mech Ageing Dev, 2002
- Insulin produced minimal activation of insulin receptor substrate IRS 1  [ **IRS1** ]  associated PI3K  [ **PIK3R1** ]  activity in fetal liver.  Ref: 12217903 Am J Physiol Endocrinol Metab, 2002
- 4 The insulin receptor substrate 1  [ **IRS1** ]  2 associated PI3K  [ **PIK3R1** ] , Shc proteins, and the kinases Akt and Erk1 2, downstream mediators of the antiapoptotic signaling by IGF IR, were activated to a higher extent and for a longer time period by ATA, compared with IGF I.  Ref: 11861505 Endocrinology, 2002
- Overexpression of a catalytically inactive dominant negative SHP 1 markedly attenuated the AT2 receptor mediated inhibition of IRS  [ **IRS1** ]  2 associated PI3K  [ **PIK3R1** ]  activity, Akt phosphorylation, and antiapoptotic effect induced by insulin.  Ref: 12198247 Mol Endocrinol, 2002
- AT2 receptor stimulation did not change insulin induced tyrosine phosphorylation of IRS  [ **IRS1** ]  2 or its association with the p85alpha subunit of PI3K  [ **PIK3R1** ] , but led to a significant reduction of insulin induced p85alpha phosphorylation.  Ref: 12198247 Mol Endocrinol, 2002
- In peripheral tissues, insulin signaling involves activation of the insulin receptor substrate IRS  [ **IRS1** ]  phosphatidylinositol 3 kinase PI3K  [ **PIK3R1** ]  enzyme system.  Ref: 12540590 Diabetes, 2003
- In the biopsies, insulin receptor kinase IRK activity, insulin receptor substrate IRS 1  [ **IRS1** ]  associated phosphatidylinositol 3 kinase PI3K  [ **PIK3R1** ]  activity, Ser 473 and Thr 308 phosphorylation of protein kinase B PKB, and protein expression of IRS 1  [ **IRS1** ] , IRS  [ **IRS1** ]  2, phosphoinositol dependent kinase 1 PDK 1, PKB, and GLUT 4 were determined.  Ref: 12196460 Diabetes, 2002
- Immunoprecipitation Western blot studies revealed that CLA inhibited IGF I induced phosphorylation of IGF IR and insulin receptor substrate IRS 1  [ **IRS1** ] , recruitment of the p85 regulatory subunit of phosphatidylinositol 3 kinase PI3K  [ **PIK3R1** ]  to IGF IR, IGF IR associated PI3K  [ **PIK3R1** ]  activity, and phosphorylated Akt and extracellular signal regulated kinase ERK 1 2 levels.  Ref: 12888657 J NutrJ Nutr, 2002
- docking of p85 alpha  [ **PIK3R1** ]  to IRS  [ **IRS1** ]  2.  Ref: 12020946 Mech Ageing Dev, 2002
- These results suggest that ceramide may induce insulin resistance via the suppression of IRS 1  [ **IRS1** ]  PI3K  [ **PIK3R1** ]  signaling, and subsequent activation of PKCzeta.  Ref: 12524657 Metabolism, 2003
- To test the hypothesis that hypothalamic insulin action involves intracellular PI3K  [ **PIK3R1** ]  signaling, we used histochemical and biochemical methods to determine the effect of insulin on hypothalamic IRS  [ **IRS1** ]  PI3K  [ **PIK3R1** ]  activity.  Ref: 12540590 Diabetes, 2003
- We investigated the effect of ceramide on insulin signaling pathways, such as insulin receptor IR beta subunit, insulin receptor substrate 1  [ **IRS1** ]  IRS 1  [ **IRS1** ] , phosphatidylinositol 3 kinase PI3K  [ **PIK3R1** ] , and protein kinase Czeta PKCzeta in rat adipocytes.  Ref: 12524657 Metabolism, 2003
- In summary, i PI3K  [ **PIK3R1** ]  mediates insulin induced reduction of IRS 1  [ **IRS1** ]  by phosphorylating it while a PI3K  [ **PIK3R1** ]  mTOR pathway controls insulin induced reduction of IRS  [ **IRS1** ]  2, ii in L6 cells, IRS  [ **IRS1** ]  2 is the major adapter molecule linking the insulin receptor to activation of PKB and MAPK, iii the mechanism of IRS 1  [ **IRS1** ]  2 down regulation is different in L6 cells compared with 3T3 L1 adipocytes.  Ref: 12594228 J Biol Chem, 2003
- Taken together, these results indicate that AT2 receptor mediated activation of SHP 1 and the consequent inhibition IRS  [ **IRS1** ]  2 associated PI3K  [ **PIK3R1** ]  activity contributed at least partly to the inhibition of Akt phosphorylation, thereby inducing apoptosis.  Ref: 12198247 Mol Endocrinol, 2002
- These findings support the hypothesis that the IRS  [ **IRS1** ]  PI3K  [ **PIK3R1** ]  pathway is a mediator of insulin action in the arcuate nucleus and, combined with recent evidence that leptin activates PI3K  [ **PIK3R1** ]  signaling in the hypothalamus, provide a plausible mechanism for neuronal cross talk between insulin and leptin signaling.  Ref: 12540590 Diabetes, 2003
- Although plasma insulin had reached 98% of its final concentration within 10 min, insulin receptor kinase IRK activity, p85 associated with insulin receptor substrate 1  [ **IRS1** ]  IRS 1  [ **IRS1** ] , IRS 1  [ **IRS1** ]  associated phosphatidylinositol 3 kinase PI3K  [ **PIK3R1** ]  activity, and Thr 308 protein kinase B PKB phosphorylation in the muscle biopsies at 20 min had reached only 6 4 and 47% respectively of those at 180 min.  Ref: 11874713 J Endocrinol, 2002
- Data presented here provide strong evidence that IGF IR deficient brown adipocytes show an increased insulin sensitivity via IRS 1  [ **IRS1** ]  Grb  [ **PIK3R1** ]  2 MAPK, resulting in an increased mitogenesis in response to insulin.  Ref: 11872675 Diabetes, 2002
- In conclusion, the reduction in IRS  [ **IRS1** ]  proteins via different PI3K  [ **PIK3R1** ]  mediated mechanisms contributes to the development of an insulin resistant state in L6 myoblasts.  Ref: 12594228 J Biol Chem, 2003
- Here we report that SP A treatment leads to rapid tyrosine specific phosphorylation of several important proteins in lung epithelial cells including insulin receptor substrate 1  [ **IRS1** ]  IRS 1  [ **IRS1** ] , an upstream activator of PI3K  [ **PIK3R1** ] .  Ref: 12413892 Exp Cell Res, 2002
- RINm5F cells overexpressing wild type GTK also exhibited reduced activation of IRS  [ **IRS1** ]  2, PI3K  [ **PIK3R1** ]  and AKT.  Ref: 12520087 Mol Med, 2002
- Thus, a prolonged activation of Erk1 2 and PI3K  [ **PIK3R1** ]  signaling pathways is important in committing a beta cell to a mitogenic event, and it is likely that this sustained activation is instigated by signal transduction occurring specifically through IRS  [ **IRS1** ]  2.  Ref: 11916914 Diabetes, 2002
- The thiazolidenedione, rosiglitazone, increases basal and or insulin stimulated glucose transport in various cell types by diverse but uncertain mechanisms that may involve insulin receptor substrate IRS 1  [ **IRS1** ]  dependent PI3K  [ **PIK3R1** ] .  Ref: 11956152 Endocrinology, 2002
- We also examined the effect of various concentrations of C 6 ceramide pretreatment on insulin induced autophosphorylation of the IR beta subunit, tyrosine phosphorylation of IRS 1  [ **IRS1** ] , enzyme activity of PI3K  [ **PIK3R1** ] , and membrane associated PKCzeta immunoreactivity.  Ref: 12524657 Metabolism, 2003
- this Cbl dependent pathway complements the IRS 1  [ **IRS1** ]  and IRS  [ **IRS1** ]  2 pathways for activating PI3K  [ **PIK3R1** ] , PDK 1, and PKC lambda during combined actions of rosiglitazone and insulin in 3T3 L1 cells.  Ref: 11956152 Endocrinology, 2002
- In this study we present data suggesting that the InR beta IRS 1  [ **IRS1** ]  PI3K  [ **PIK3R1** ]  pathway is attenuated in the Snell dwarf mouse liver.  Ref: 12020946 Mech Ageing Dev, 2002
- Leptin stimulation activates JAK STAT, IRS 1  [ **IRS1** ]  PI3K  [ **PIK3R1** ]  and MAPK signalling pathways.  Ref: 12823272 Clin Exp Immunol, 2003
- Insulin receptor substrate IRS 1  [ **IRS1** ]  was associated with PI3K  [ **PIK3R1** ]  activity, which is necessary for Akt activation by insulin, and was reduced in obese muscles, and this was accompanied by decreased IRS 1  [ **IRS1** ]  expression.  Ref: 12663464 Diabetes, 2003
- These include a decreased IRS  [ **IRS1** ]  2 pool level, a decrease in PI3K  [ **PIK3R1** ]  activity and its association with IRS  [ **IRS1** ]  2 and decreased docking of p85alpha to IRS  [ **IRS1** ]  2.  Ref: 12020945 Mech Ageing Dev, 2002
- While IGF I induced activation of IRS 1  [ **IRS1** ] , Shc, PI3K  [ **PIK3R1** ] , and MAPK pathways was unaffected, IGF I inducible beta1 integrin associated kinase activity and association of Crk with p130 were significantly inhibited by RACK1 overexpression.  Ref: 11884618 Mol Cell Biol, 2002
- Additionally, rosiglitazone increased IRS 1  [ **IRS1** ]  and IRS  [ **IRS1** ]  2 levels, thereby enhancing insulin effects on IRS 1  [ **IRS1** ]  and IRS  [ **IRS1** ]  2 dependent PI3K  [ **PIK3R1** ]  and downstream signaling factors PKC lambda and protein kinase B.  Ref: 11956152 Endocrinology, 2002
- Our data also suggest a preferential docking of IRS  [ **IRS1** ]  2 p85 alpha  [ **PIK3R1** ]  p110 alpha in the aged dwarf liver and IRS  [ **IRS1** ]  2 p85 alpha  [ **PIK3R1** ]  p110 beta in the aged control.  Ref: 12020945 Mech Ageing Dev, 2002
- Downstream, the association IRS 1  [ **IRS1** ]  growth factor receptor binding protein 2 Grb  [ **PIK3R1** ]  2 was augmented in the IGF IR brown adipocyte cell line.  Ref: 11872675 Diabetes, 2002
- Conversely, the disappearance of the IRS 1  [ **IRS1** ]  PI3K  [ **PIK3R1** ]  complex from this fraction has been linked to insulin desensitization.  Ref: 11875115 Mol Endocrinol, 2002
- However, increased IRS  [ **IRS1** ]  3 expression blocked glucose IGF 1 induced IRS  [ **IRS1** ]  2 translocation from the cytosol to the plasma membrane, dampening IRS  [ **IRS1** ]  2 IGF 1R interaction and subsequent activation of the PI3K  [ **PIK3R1** ]  PKB GSK3 signaling pathway.  Ref: 12850284 Mol Cell Endocrinol, 2003
- Therefore, we have characterized survival enhancing signaling activated by SP A leading from SPAR through IRS 1  [ **IRS1** ] , PI3K  [ **PIK3R1** ] , PKB Akt, and FKHR.  Ref: 12413892 Exp Cell Res, 2002
- ... IRS 1  [ **IRS1** ]  associated PI3K  [ **PIK3R1** ]  activity was not significantly influenced.  Ref: 12198247 Mol Endocrinol, 2002
- IRS  [ **IRS1** ]  2 and phosphotyrosine associated PI3K  [ **PIK3R1** ]  activity were increased 3.5 and 4.8 fold, respectively, by insulin in glycerol infused rats but only 1.6 and 2.3 fold in lipid infused rats.  Ref: 12095990 J Biol Chem, 2002
- but not accompanied by increases in IRS 1  [ **IRS1** ]  2 dependent PI3K  [ **PIK3R1** ]  or protein kinase B activity.  Ref: 11956152 Endocrinology, 2002
- These data emphasize the importance of IRS  [ **IRS1** ]  2 PI3K  [ **PIK3R1** ]  PKB signal transduction for beta cell growth and survival.  Ref: 12850284 Mol Cell Endocrinol, 2003
- We then identified an increase in JAK2,  **IRS1**  and IRS2 tyrosine phosphorylation and in their association with p85, a subunit of PI3K  [ **PIK3R1** ] .  Ref: 12706299 Mol Cell Endocrinol, 2003
- This was accompanied by reduced expression of IRS  [ **IRS1** ]  2 and attenuated insulin stimulated recruitment of PI3K  [ **PIK3R1** ]  to IRS 1  [ **IRS1** ]  and IRS  [ **IRS1** ]  2, yet insulin stimulated PKB activity and phosphorylation of eNOS at Ser1177 were unaffected.  Ref: 12644458 J Biol Chem, 2003
- Insulin stimulated IRS 1  [ **IRS1** ]  associated PI3K  [ **PIK3R1** ]  activity in muscle of glycerol infused rats 2.4 fold but had no effect in lipid infused rats.  Ref: 12095990 J Biol Chem, 2002
- At the molecular level, impaired activation of the IR IRS  [ **IRS1** ]  PI3K  [ **PIK3R1** ]  pathway and a significant decrease both in the levels and in insulin stimulated activation of the serine threonine kinase Akt were observed.  Ref: 12767044 J Cell Physiol, 2003
- Pretreatment with C 6 ceramide significantly reduced autophosphorylation of the IR beta subunit, tyrosine phosphorylation of IRS 1  [ **IRS1** ] , and enzyme activity of PI3K  [ **PIK3R1** ] .  Ref: 12524657 Metabolism, 2003
